# Whole-genome sequencing of SARS-CoV-2 in Uganda: implementation of the low-cost ARTIC protocol in resource-limited settings

**DOI:** 10.12688/f1000research.53567.1

**Published:** 2021-07-19

**Authors:** Gerald Mboowa, Savannah Mwesigwa, David Kateete, Misaki Wayengera, Emmanuel Nasinghe, Eric Katagirya, Ashaba Fred Katabazi, Edgar Kigozi, Samuel Kirimunda, Rogers Kamulegeya, Jupiter Marina Kabahita, Moses Nsubuga Luutu, Patricia Nabisubi, Stephen Kanyerezi, Bernard Ssentalo Bagaya, Moses L Joloba

**Affiliations:** 1 Immunology and Molecular Biology, Makerere University, Kampala, Uganda; 2 The African Center of Excellence in Bioinformatics and Data-Intensive Sciences, The Infectious Diseases Institute, Kampala, Uganda; 3 The Supranational Tuberculosis Reference Laboratory, Uganda National Health Laboratories - UNHLS, Luzira, Uganda

**Keywords:** COVID-19, Makerere University, Uganda, SARS-CoV-2, Whole-genome sequencing, PRESIDE, RT-PCR

## Abstract

**Background:** In January 2020, a previously unknown coronavirus strain was identified as the cause of a severe acute respiratory syndrome (SARS-CoV-2). The first viral whole-genome was sequenced using high-throughput sequencing from a sample collected in Wuhan, China. Whole-genome sequencing (WGS) is imperative in investigating disease outbreak transmission dynamics and guiding decision-making in public health.

**Methods:** We retrieved archived SARS-CoV-2 samples at the Integrated Biorepository of H3Africa Uganda, Makerere University (IBRH3AU). These samples were collected previously from individuals diagnosed with coronavirus disease 2019 (COVID-19) using real-time reverse transcription quantitative polymerase chain reaction (RT-qPCR). 30 samples with cycle thresholds (Cts) values <25 were selected for WGS using SARS-CoV-2 ARTIC protocol at Makerere University Molecular Diagnostics Laboratory.

**Results:** 28 out of 30 (93.3%) samples generated analyzable genomic sequence reads. We detected SARS-CoV-2 and lineages A (22/28) and B (6/28) from the samples. We further show phylogenetic relatedness of these isolates alongside other 328 Uganda (lineage A = 222, lineage B = 106) SARS-CoV-2 genomes available in GISAID by April 22, 2021 and submitted by the Uganda Virus Research Institute.

**Conclusions:** Our study demonstrated adoption and optimization of the low-cost ARTIC SARS-CoV-2 WGS protocol in a resource limited laboratory setting. This work has set a foundation to enable rapid expansion of SARS-CoV-2 WGS in Uganda as part of the Presidential Scientific Initiative on Epidemics (PRESIDE) CoV-bank project and IBRH3AU.

## Introduction

The severe acute respiratory syndrome coronavirus 2 (SARS-CoV-2) causes coronavirus disease 2019 (COVID-19) which has now spread throughout the entire world, causing more than 175 million infections and over 3.7 million deaths globally. ^1^ During early 2020, whole-genome sequencing (WGS) enabled researchers to rapidly identify SARS-CoV-2, and knowing the genome sequence allowed rapid development of diagnostic tests and other appropriate tools needed for the response to this novel infection. Continued genome sequencing supports the monitoring of the disease’s spread, activity, viral evolution as well as emerging new viral variants. ^2^ The COVID-19 pandemic is still ongoing and the global response will have to continue for the foreseeable future; the World Health Organization (WHO) has recommended WGS to be further adopted as well as implemented in new settings and new uses to better understand the world of emerging pathogens and their interactions with humans in a variety of climates, ecosystems, cultures, lifestyles, biomes ^2^ and genetic backgrounds.

Uganda has had approximately 60,250 COVID-19 cases and 423 deaths by June 12, 2021. ^3^ As more countries move to implement genome sequencing programmes, Uganda is among those embracing SARS-CoV-2 WGS. Of the 131 Uganda full SARS-CoV-2 genomes analysed in December 2020, 50 (38%) belonged to lineage A and the rest belonged to a variety of B lineages with the majority lineages being B.1 (N = 30; 23%) and B.1.5 (N = 17; 13%) which were found predominantly in cross border truck drivers seeking to enter the country. ^4^ As of April 26, 2021, a total of 328 SARS-CoV-2 samples had been sequenced and deposited in the GISAID ^5^ by the Uganda Virus Research Institute (UVRI). ^6^ This represented 0.8% of the total COVID-19 cases that had been detected in the country at that time. This situation is very similar to almost all other African countries, yet this ongoing global pandemic has already demonstrated the importance of widespread access to rapid novel pathogen discovery and subsequent surveillance, as well as comprehensive pathogen information sharing.

We piloted sequencing of SARS-CoV-2 samples at the Molecular Diagnostics Laboratory located in the Department of Immunology and Molecular Biology, Makerere University for two reasons: (i) to test the feasibility of ARTIC amplicon-based genome sequencing at our local institution; and (ii) to extend genomic analyses for COVID-19 surveillance in Uganda. ARTIC protocol was selected due to its low cost and high sensitivity, as well as its scalability compared to other sequencing methods. ^7^ Routine SARS-CoV-2 genome sequencing in many places still faces difficulties such as an unreliable supply-chain for WGS reagents, since many of these are imported from western countries, limited technical expertise as well as genomic infrastructure, and relatively high costs of genome sequencing. Consequently, this work also served as a feasibility study to assess the implementation, practicality, and adoption of ARTIC amplicon-based sequencing in Ugandan’s resource-limited settings, allowing future efforts to integrate and expand into routine laboratory diagnostic pipelines. This current study served as a proof-of-concept to extend genomic capacity for COVID-19 surveillance at Makerere University, College of Health Sciences (MakCHS) Molecular Diagnostic Laboratory. This laboratory has been performing SARS-CoV-2 reverse transcription quantitative polymerase chain reaction (RT-qPCR) since March 2020 and was among the first facilities in Uganda to be accredited by the Ministry of Health to carry out routine SARS-CoV-2 testing. We sought to perform WGS of 30 SARS-CoV-2 RT-qPCR positive samples using the COVID-19 ARTIC v3 amplicon-based sequencing protocol in our settings using the Illumina MiSeq sequencing platform. We considered samples based on the criteria below;

- Cycle thresholds (Cts) values below 25. This is because the ARTIC SARS-CoV-2 sequencing protocol produces longer and high-quality genomes with Ct values below 30. ^8^
- We sequenced samples collected after September 2020 to increase the chances of detecting any of the emerging circulating SARS-CoV-2 variants including the high mortality ^9^ variant originally referred to as the UK variant or B.1.1.7, and the highly transmissible ^10^ variant originally referred to as the South Africa variant or B.1.351/501Y.V2 which had been reported on 14 December and 18 December, 2020 respectively. ^11^
- All our sequenced isolates in this study were therefore selected from those archived samples collected after September 2020.

## Methods

### Ethical consideration

The Integrated Biorepository of H3Africa Uganda (IBRH3AU) received ethical approval from Makerere University School of Biomedical Sciences Research and Ethics Committee (SBS-REC) and from the Uganda National Council for Science and Technology (UNCST) to collect, process, store, and share biospecimens including COVID-19 specimens. Additionally, the IBRH3AU obtained ethical approval from the Mulago National Hospital REC (Protocol Number MHREC 1868 and approved on March 27 ^th^, 2021). Participants consented in writing to sample storage and subsequent use of their samples in current and future studies related to understanding SARS-CoV-2 infection in Uganda.

### Study design

This was a cross-sectional study design.

### Study settings

Samples for this study included nasopharyngeal swabs collected from individuals who had a positive COVID-19 test at the Molecular Diagnostic Laboratory. COVID-19 samples processed at this facility were archived at the Integrated Biorepository of H3Africa Uganda – IBRH3AU. These samples were collected between September 2020 and February 2021 from individuals coming from Kampala metropolitan area, which consists of Kampala city itself and the neighboring Wakiso, Mukono, Mpigi, Buikwe and Luweero districts of Uganda.

### Sample preparation and nucleic acid extraction

Total nucleic acid was extracted using the QIAamp Viral RNA Mini Kit (Qiagen) at the MakCHS Molecular Diagnostic Laboratory, as per the manufacturer’s protocol. All patient samples had initially been assessed by RT-qPCR for SARS-CoV-2 viral RNA using a triplex approach that targets the *N*, *ORF* and *S* viral genes. Therefore, this study used samples that were diagnostically SARS-CoV-2 positive with amplification of the targeted region(s) crossing the threshold before 25 PCR cycles. In total, 30 SARS-CoV-2 positive samples were selected randomly for high-throughput genome sequencing at our facility using a MiSeq Illumina platform.

### Sequencing and bioinformatics analysis

These samples had been collected and previously stored from patients diagnosed with COVID-19 using real-time RT-qPCR. Metadata associated with the patient samples included the date of sample collection, gender, nationality, and purpose of testing (Routine, Contact, Alert, Travelers, Quarantine, Case, and Professional jobs requiring COVID-19 PCR test). ARTIC amplicon-based sequencing was used to generate 400 bp amplicons with 75 bp overlaps covering the length of the ~ 29.9 kB SARS-CoV-2 genome as described elsewhere. ^12^ Briefly, cDNA synthesis was carried out with random primers (Protoscript II First Strand cDNA Synthesis Kit, E6560S) followed by PCR amplification using ARTIC primers. Genomic library preparation was carried out using the Nextera XT DNA Library Preparation kit (15032355) according to manufactures' recommendations, and sequencing was carried out on the Illumina MiSeq platform (Illumina, CA, USA) using MiSeq Reagent Kit v3 (600-cycle, #MS-102-3003), according to manufacturer’s protocol.

Quality control (QC) was carried out before viral genome fasta generation, as previously described. ^13^ Briefly, demultiplexed fastq files generated by sequencing were used as an input data for the analysis. Reads were trimmed based on quality scores with a cutoff threshold of Q30 to remove low-quality regions, in addition to adapter sequences. QC assessment for sequence reads was performed using FastQC (v0.11.9) ^14^ and MultiQC (v1.9). ^15^

For those reads passing the QC cutoff, we used Pangolin COVID-19 lineage assigner (v3.0.5) ^16^ to assign SARS-CoV-2 viral lineages. Phylogenetic analysis was carried out in order to understand the evolution of this virus within the Ugandan population, including other SARS-CoV-2 genomes from Uganda that had been submitted by the Uganda Virus Research Institute (UVRI) to the GISAID database by April 22, 2021, and only complete sequences were included, totaling 328 SARS-CoV-2 genomes.

**Figure 1.**
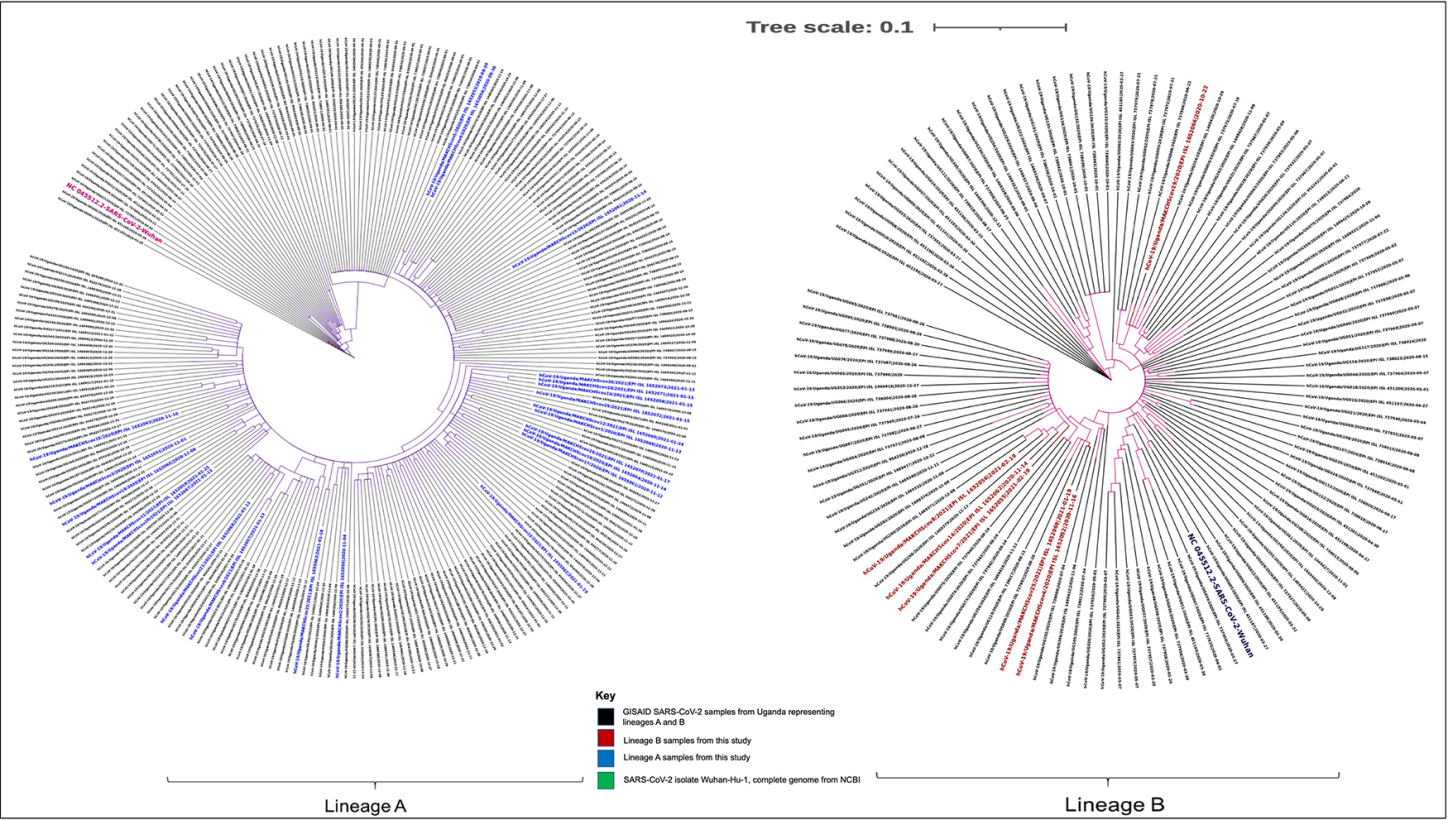
Phylogenetic analysis of 356 SARS-CoV-2 genome isolates (Lineages A and B) from Uganda.

**Figure 2.**
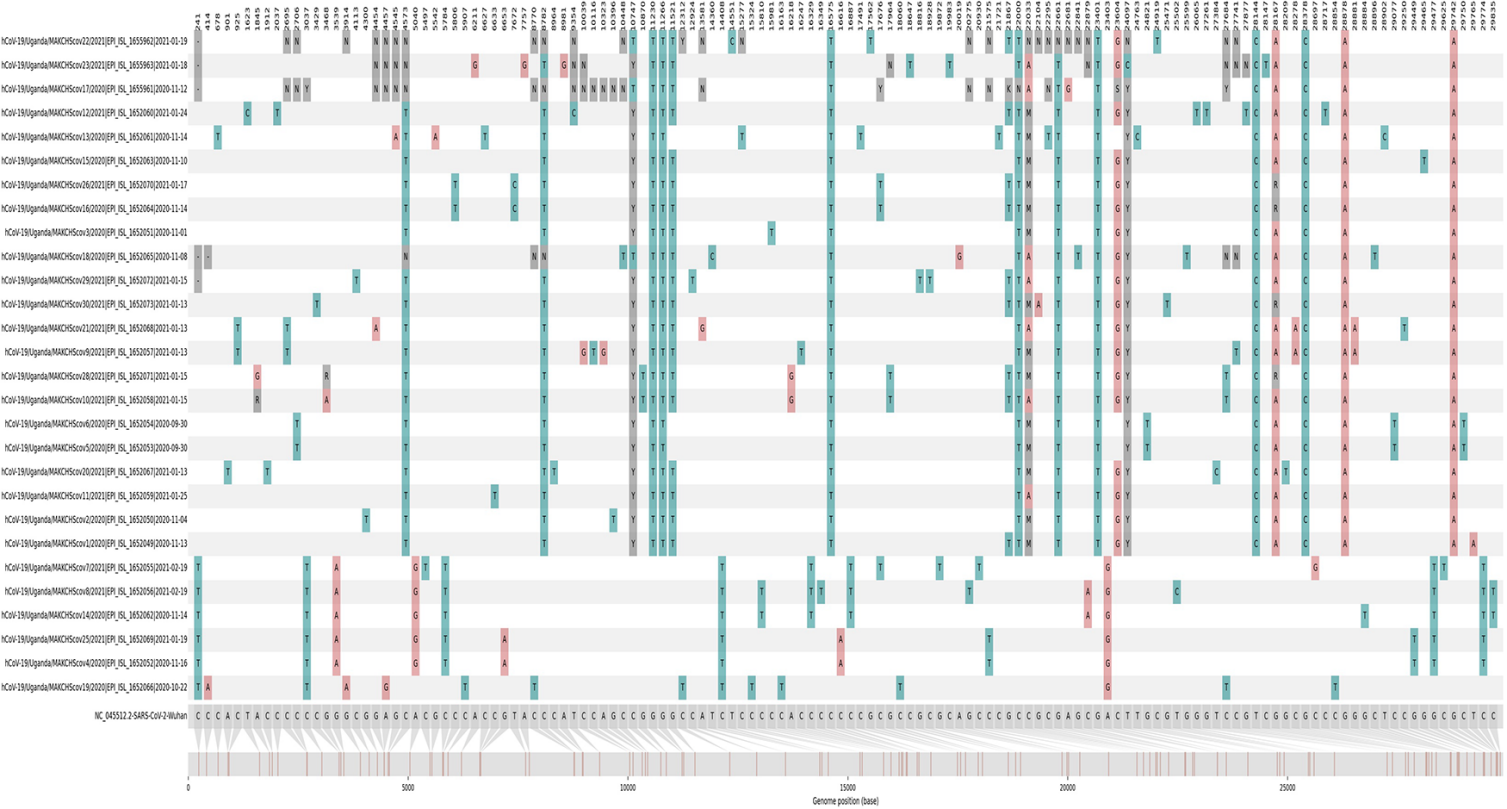
Nucleotide alignment showing variants covering the SARS-CoV-2 sample genome lengths.

Multiple sequence alignment of 328 Ugandan SARS-CoV-2 genomes and 28 from MakCHS Molecular Diagnostics Laboratory was performed using the web version of MAFFT v.7.475. ^17^ In each alignment, the SARS-CoV-2 reference sequence (NCBI Reference Sequence: SARS-CoV-2 isolate Wuhan-Hu-1, complete genome, NC_045512.2) was included. The alignment from MAFFT was then subjected to snp-sites v2.3.3 ^18^ to generate a phylip file format, which was later used to infer a maximum likelihood tree using PhyML v3.3.3 ^19^ with the tool’s default parameters. The tree generated by PhyML was stored in a newick file format. The file was then uploaded to the interactive tree of life ( iTOL v4.0) ^20^ - an online tool for phylogenetic tree display and annotation for visualization ( Figure 1). We then used snipit v1.0.3 ^21^ to zoom into the 28 genomes from MakCHS sequencing lab to visualize their snps in reference to the SARS-CoV-2 reference genome ( Figure 2).

## Results

### COVID-19 samples

The average age of the study participants was 40 years with an equal ratio of males to females. Their nationalities included 21 Ugandans, 3 Eritreans, 2 Indians, 1 Israeli and 1 South Sudanese. After WGS, we successfully generated a total of 28 out 30 (93%) analyzable SARS-CoV-2 genomes. It was probable that only these archived specimens had adequate viral RNA for successful genomic sequencing. Targeted SARS-CoV-2 RT-qPCR was positive for *N*, *ORF*, and *S* viral genes, however two samples did not have detectable Ct values for viral *S* gene.

### Sequencing quality

Both patient demographics and summary characteristics of these genomes are shown in Table 1. We found more A strains of SARS-CoV-2 than B strains. The different quality metrics used included SARS-CoV-2 draft genome length, GC-content and average depth of coverage. The coverage of all samples was above 30X.

**Table 1.** Summary characteristics of SARS-CoV-2 genomes obtained from 28 individuals from IBR3HAU.

Sample_ID	Genome length	GC-Content	Depth	Sample collection date	Patient age (y)	Gender	Category	CT value			Lineage
								N	ORF	S	
hCoV-19/Uganda/MAKCHScov1/2020	29135	37.95	621X	2020-11-13	34	M	-	16.43	16.42	17.08	A.23.1
hCoV-19/Uganda/MAKCHScov2/2020	29592	37.96	735X	2020-11-04	49	F	-	15.84	15.74	16.23	A.23.1
hCoV-19/Uganda/MAKCHScov3/2020	29340	37.80	1132X	2020-11-01	43	M	-	15.21	15.07	15.44	A.23.1
hCoV-19/Uganda/MAKCHScov4/2020	29284	37.81	601X	2020-11-16	49	F	-	15.84	15.74	16.23	B.1.393
hCoV-19/Uganda/MAKCHScov5/2020	29654	37.96	716X	2020-09-30	41	F	-	15.86	17.44	18.65	A
hCoV-19/Uganda/MAKCHScov6/2020	28944	38.03	668X	2020-09-30	50	M	-	15.89	15.89	-	A
hCoV-19/Uganda/MAKCHScov7/2021	29561	37.95	1220X	2021-02-19	34	F	-	16.07	17.21	17.59	B.1.393
hCoV-19/Uganda/MAKCHScov8/2021	28944	38.03	668X	2021-02-19	34	M	-	16.15	17.62	-	B.1.258
hCoV-19/Uganda/MAKCHScov9/2021	29592	37.96	733X	2021-01-13	30	F	-	19.34	19.99	20.80	A.23.1
hCoV-19/Uganda/MAKCHScov10/2021	29374	37.92	639X	2021-01-15	49	F	Alert	17.55	18.20	18.97	A.23.1
hCoV-19/Uganda/MAKCHScov11/2021	28435	38.07	629X	2021-01-25	36	F	Contact	20.47	20.21	20.67	A.23.1
hCoV-19/Uganda/MAKCHScov12/2021	28548	38.07	775X	2021-01-24	58	M	-	16.59	16.82	17.26	A.23.1
hCoV-19/Uganda/MAKCHScov13/2020	29185	38.00	637X	2020-11-14	24	M	-	17.02	16.86	17.37	A
hCoV-19/Uganda/MAKCHScov14/2020	27826	38.00	660X	2020-11-14	20	F		17.13	16.93	-	B.1.258
hCoV-19/Uganda/MAKCHScov15/2020	27470	38.01	652X	2020-11-10	46	M	-	17.31	16.69	17.22	A.23.1
hCoV-19/Uganda/MAKCHScov16/2020	29666	37.97	611X	2020-11-14	36	F	-	17.55	17.42	17.99	A.23.1
hCoV-19/Uganda/MAKCHScov17/2020	213211	45.96	92X	2020-11-12	49	F	-	17.55	18.20	18.97	A.23
hCoV-19/Uganda/MAKCHScov18/2020	27470	38.01	296X	2020-11-08	41	M	-	18.19	17.57	18.31	A.23.1
hCoV-19/Uganda/MAKCHScov19/2020	29093	37.72	606X	2020-10-22	60	M		18.23	18.56	19.04	B.1.214
hCoV-19/Uganda/MAKCHScov20/2021	28006	38.06	656X	2021-01-13	29	M	-	19.06	19.59	20.33	A.23.1
hCoV-19/Uganda/MAKCHScov21/2021	28910	37.98	730X	2021-01-13	28	M	-	19.34	19.99	20.79	A.23.1
hCoV-19/Uganda/MAKCHScov22/2021	55370	40.59	316X	2021-01-19	31	F	-	19.58	19.74	20.13	A.23.1
hCoV-19/Uganda/MAKCHScov23/2021	44685	42.04	262X	2021-01-18	63	F	-	19.80	21.04	20.54	A.23.1
hCoV-19/Uganda/MAKCHScov25/2021	29262	38.02	670X	2021-01-19	45	M	Routine	16.46	16.83	17.13	B.1.393
hCoV-19/Uganda/MAKCHScov26/2021	29666	37.97	612X	2021-01-17	33	M	Routine	13.40	13.97	14.28	A.23.1
hCoV-19/Uganda/MAKCHScov28/2021	29592	37.96	735X	2021-01-15	35	M	-	22.89	22.73	23.09	A.23.1
hCoV-19/Uganda/MAKCHScov29/2021	29662	37.94	663X	2021-01-15	33	F	-	23.24	23.29	23.72	A.23.1
hCoV-19/Uganda/MAKCHScov30/2021	28078	38.09	634X	2021-01-13	46	F	-	23.53	26.48	26.82	A.23.1

### Phylogenetic analysis

Of the virus genomes generated, we were unable to identify any known SARS-CoV-2 variants of concern or interest from the sampled specimens when we compared the genomes from specimens collected in September 2020 to February 2021. Phylogenetic analysis of all the sequenced SARS-CoV-2 genomes by then from Uganda shows genomic relatedness as seen in Figure 1; however, this observation was of limited value given that there was inadequate epidemiologic data from the patients from whom these specimens had been collected. There was close genomic relatedness detected in MAKCHScov28 and cov10 as well as MAKCHScov5 and cov6 that had been collected from different patients on the same date. MAKCHScov26 and cov16 as well as MAKCHScov25 and cov4 were equally closely linked through phylogenetic analysis, though collected at least two months apart. Hence, this phylogenetic relatedness is likely to indicate local transmission events of SARS-CoV-2 in Kampala metropolitan area.

## Discussion

Due to the fact that 28/30 of SARS-CoV-2 genomes were successfully sequenced on the MiSeq platform, we have demonstrated a proof-of-concept project on SARS-CoV-2 WGS using archived clinical nasopharyngeal swab samples from the IBRH3AU. This study has successfully optimized and validated SARS-CoV-2 WGS using the ARTIC amplicon sequencing protocol. This has enabled us to adopt SARS-CoV-2 WGS at MakCHS Molecular Diagnostic Laboratory. As of June 8, 2021, Uganda had registered 53,961 COVID-19 cases and more than half (~26,000) of the samples are stored at the IBR3HAU biorepository.

Globally, researchers are being encouraged to sequence and share more genomes of SARS-CoV-2 via the GISAID platform, and there are currently more than 1.8 million coronavirus genome sequences from 172 countries and territories, which is a great testament to the hard work of researchers around the world during the COVID-19 global pandemic. ^22^ Ironically, approximately 98% of these genomes have been submitted by high-income countries, underscoring the need to build a similar capacity in lower-middle-income settings (LIMC).

In this study, we estimated that the cost of WGS per SARS-CoV-2 clinical specimen in our laboratory to be $110 compared to $57.87 ^23^ in the United States using a multiplex PCR followed by sequencing on an Illumina MiSeq apparatus. In the United Arab Emirates, the cost of SARS-CoV-2 full genome sequencing was estimated to be ~$87 per specimen when sequencing 96 samples in a batch at 400× using the target enrichment method. ^24^ It should be noted that target enrichment sequencing is still a more cost-effective approach and is scalable in many settings that handle large volumes of these samples. As of June 8, 2021, GISAID had 1,885,406 hCoV-19 genomic data with only a total of 19,065 submissions being from Africa while on June 1 2021, a total of 4,843,874 COVID-19 cases and 130,814 deaths (CFR: 2.7%) had been reported in 55 African Union (AU) member states representing 3% of all cases reportedly globally. ^25^ However, a majority of the low-quality SARS-CoV-2 genomes submitted in this same online genome database have been submitted from sequencing facilities in Africa.

As many countries and territories globally continue to find the optimal approach in managing the health-related consequences of COVID-19, more laboratories in these settings must find the best or affordable protocols to implement WGS of SARS-CoV-2 to inform public health measures. We performed WGS of 30 samples and specifically successfully evaluated the performance of ARTIC SARS-CoV-2 sequencing protocol performance in our settings using the Illumina MiSeq platform. In this study, genomic libraries were generated using RNA samples isolated from either newly prepared nasopharyngeal swabs in AVL buffer, which is a lysis buffer intended for purifying viral nucleic acids, or previously collected and frozen nasopharyngeal swabs preserved in AVL buffer. Therefore, the findings of this study offer guidance on implementing the low-cost ARTIC SARS-CoV-2 genome sequencing protocol to study SARS-CoV-2 genomic variations in resource limited settings. Many of these settings are currently unable to perform real-time WGS of such samples either due to absence of sequencing infrastructure, which in some cases has been overcome by establishing collaborations with sequencing facilities in other countries and therefore requiring shipment of the samples, unsustainable supply of sequencing reagents, or lack of trained genomics and bioinformatics personnel. WGS of SARS-CoV-2 remains vital in elucidating COVID-19 disease ^26^ for the unforeseeable future as researchers globally continue to identify new SARS-CoV-2 variants of concern and interests such as B.1.1.7 (Alpha), B.1351 (Beta), P.1 (Gamma), B.1.617.2 (Delta) and B.1.427/B.1.429 (Epsilon), P.2 (Zeta), B.1.525 (B.1.525), P.3 (Theta), B.1.526 (Iota), and B.1.617.1 (Kappa) respectively. ^27^ WGS allows detection and characterization of these emerging viral variants, generating essential new information about their genomic, immunologic, virologic, epidemiologic, and clinical characteristics.

Incomplete epidemiological and clinical characteristics as well as lack of COVID-19 disease severity of study participants are some of the limitations of this study. Also, the small sample size used as well as sequencing of samples that had been collected from Kampala metropolitan area may not represent the true proportion of identified SARS-CoV-2 lineages and variants in Uganda during the study period.

We recommend the establishment of more collaborative consortia between researchers, as well between National Public Health Institutions in LIMCs and developed countries to build low-cost, sustainable, functioning pathogen genome sequencing facilities to accelerate pathogen discovery and outbreak surveillance using WGS. Furthermore, these facilities can equally utilize recently developed multiplex RT-qPCR assays to screen for SARS-CoV-2 variants of concern or interest and monitor their frequencies. These variant genotyping assays not only complement WGS in such settings but also offer a cost-effective way to identify which samples can be prioritized for WGS, especially those that are unidentifiable by routine genotyping tests. Even during the vaccination phase of COVID-19, documenting incidences and prevalence of these SARS-CoV-2 re-or-emerging variants is key in identifying vaccine escape mutants. This offers the opportunity for judicious use of WGS for rapid discovery of novel SARS-CoV-2 variants.

## Conclusion

In conclusion, our proof-of-concept study shows that ARTIC SARS-CoV-2 sequencing protocol on Illumina MiSeq is sensitive and accurate at higher SARS-Cov-2 template concentration (e.g., Ct value <25) in the Ugandan settings. We successfully validated the protocol and evaluated the process in mappability, genome length, GC-content, viral genome coverage, and variations in SNV calling. The result of our study provides a thorough affirmation of carrying out whole-genome sequencing for clinical SARS-CoV-2 samples in resource limited settings, thereby providing information to mitigate the impact of COVID-19 on our society. We have further contributed to SARS-CoV-2 global dataset.

## Author contributions

MLJ conceived and designed the study. EN and RK helped the patient nasopharyngeal swab sample collection. DPK, BSB, MW, ML, JMK, EK, SK and AFK performed experiments including RNA isolation, qRT-PCR, SARS-CoV-2 WGS library construction and sequencing. GM, SM, PN, SK and EK performed bioinformatics data analyses and drafted the manuscript. EN helped coordinate the project meetings and IRB application. All authors reviewed the final manuscript.

## Data Availability

These samples are available in GenBank: GenBank: Severe acute respiratory syndrome coronavirus 2 isolate SARS-CoV-2/human/UGA/MAKCHScov2/2020, complete genome. Accession number MZ287347; https://www.ncbi.nlm.nih.gov/nuccore/MZ287347 GenBank: Severe acute respiratory syndrome coronavirus 2 isolate SARS-CoV-2/human/UGA/MAKCHScov14/2020 ORF1ab polyprotein (ORF1ab), ORF1a polyprotein (ORF1ab), surface glycoprotein (S), ORF3a protein (ORF3a), envelope protein (E), membrane glycoprotein (M), ORF6 protei... Accession number: MZ287348; https://www.ncbi.nlm.nih.gov/nuccore/MZ287348 GenBank: Severe acute respiratory syndrome coronavirus 2 isolate SARS-CoV-2/human/UGA/MAKCHScov21/2021 ORF1ab polyprotein (ORF1ab), ORF1a polyprotein (ORF1ab), surface glycoprotein (S), ORF3a protein (ORF3a), envelope protein (E), membrane glycoprotein (M), ORF6 protei... Accession number: MZ287349; https://www.ncbi.nlm.nih.gov/nuccore/MZ287349 GenBank: Severe acute respiratory syndrome coronavirus 2 isolate SARS-CoV-2/human/UGA/MAKCHScov21/2021 ORF1ab polyprotein (ORF1ab), ORF1a polyprotein (ORF1ab), surface glycoprotein (S), ORF3a protein (ORF3a), envelope protein (E), membrane glycoprotein (M), ORF6 protei... Accession number: MZ287349 https://www.ncbi.nlm.nih.gov/nuccore/MZ287349 GenBank: Severe acute respiratory syndrome coronavirus 2 isolate SARS-CoV-2/human/UGA/MAKCHScov6/2020, complete genome. Accession number: MZ287350 https://www.ncbi.nlm.nih.gov/nuccore/MZ287350 GenBank: Severe acute respiratory syndrome coronavirus 2 isolate SARS-CoV-2/human/UGA/MAKCHScov11/2021 ORF1ab polyprotein (ORF1ab), ORF1a polyprotein (ORF1ab), surface glycoprotein (S), ORF3a protein (ORF3a), envelope protein (E), membrane glycoprotein (M), ORF6 protei... Accession number: MZ287351 https://www.ncbi.nlm.nih.gov/nuccore/MZ287351 GenBank: Severe acute respiratory syndrome coronavirus 2 isolate SARS-CoV-2/human/UGA/MAKCHScov30/2021, complete genome. Accession number: MZ287352 https://www.ncbi.nlm.nih.gov/nuccore/MZ287352 GenBank: Severe acute respiratory syndrome coronavirus 2 isolate SARS-CoV-2/human/UGA/MAKCHScov18/2020 ORF1ab polyprotein (ORF1ab) and ORF1a polyprotein (ORF1ab) genes, partial cds; surface glycoprotein (S), ORF3a protein (ORF3a), envelope protein (E), membrane glycopr... Accession number: MZ287353 https://www.ncbi.nlm.nih.gov/nuccore/MZ287353 GenBank: Severe acute respiratory syndrome coronavirus 2 isolate SARS-CoV-2/human/UGA/MAKCHScov26/2021, complete genome. Accession number: MZ287354 https://www.ncbi.nlm.nih.gov/nuccore/MZ287354 GenBank: Severe acute respiratory syndrome coronavirus 2 isolate SARS-CoV-2/human/UGA/MAKCHScov20/2021 ORF1ab polyprotein (ORF1ab), ORF1a polyprotein (ORF1ab), surface glycoprotein (S), ORF3a protein (ORF3a), envelope protein (E), membrane glycoprotein (M), ORF6 protei... Accession number: MZ287355 https://www.ncbi.nlm.nih.gov/nuccore/MZ287355 GenBank: Severe acute respiratory syndrome coronavirus 2 isolate SARS-CoV-2/human/UGA/MAKCHScov13/2020 ORF1ab polyprotein (ORF1ab), ORF1a polyprotein (ORF1ab), surface glycoprotein (S), ORF3a protein (ORF3a), envelope protein (E), membrane glycoprotein (M), ORF6 protei... Accession number: MZ287356 https://www.ncbi.nlm.nih.gov/nuccore/MZ287356 GenBank: Severe acute respiratory syndrome coronavirus 2 isolate SARS-CoV-2/human/UGA/MAKCHScov29/2021 ORF1ab polyprotein (ORF1ab) and ORF1a polyprotein (ORF1ab) genes, partial cds; and surface glycoprotein (S), ORF3a protein (ORF3a), envelope protein (E), membrane gly... Accession number: MZ287357 https://www.ncbi.nlm.nih.gov/nuccore/MZ287357 GenBank: Severe acute respiratory syndrome coronavirus 2 isolate SARS-CoV-2/human/UGA/MAKCHScov4/2020, complete genome. Accession number: MZ287358 https://www.ncbi.nlm.nih.gov/nuccore/MZ287358 GenBank: Severe acute respiratory syndrome coronavirus 2 isolate SARS-CoV-2/human/UGA/MAKCHScov28/2021, complete genome. Accession number: MZ287359 https://www.ncbi.nlm.nih.gov/nuccore/MZ287359 GenBank: Severe acute respiratory syndrome coronavirus 2 isolate SARS-CoV-2/human/UGA/MAKCHScov19/2020, complete genome. Accession number: MZ287360 https://www.ncbi.nlm.nih.gov/nuccore/MZ287360 GenBank: Severe acute respiratory syndrome coronavirus 2 isolate SARS-CoV-2/human/UGA/MAKCHScov7/2021, complete genome. Accession number: MZ287361 https://www.ncbi.nlm.nih.gov/nuccore/MZ287361 GenBank: Severe acute respiratory syndrome coronavirus 2 isolate SARS-CoV-2/human/UGA/MAKCHScov12/2021, complete genome. Accession number: MZ287362 https://www.ncbi.nlm.nih.gov/nuccore/MZ287362 GenBank: Severe acute respiratory syndrome coronavirus 2 isolate SARS-CoV-2/human/UGA/MAKCHScov5/2020, complete genome. Accession number: MZ287363 https://www.ncbi.nlm.nih.gov/nuccore/MZ287363 GenBank: Severe acute respiratory syndrome coronavirus 2 isolate SARS-CoV-2/human/UGA/MAKCHScov1/2020, complete genome. Accession number: MZ287364 https://www.ncbi.nlm.nih.gov/nuccore/MZ287364 GenBank: Severe acute respiratory syndrome coronavirus 2 isolate SARS-CoV-2/human/UGA/MAKCHScov15/2020 ORF1ab polyprotein (ORF1ab), ORF1a polyprotein (ORF1ab), surface glycoprotein (S), ORF3a protein (ORF3a), envelope protein (E), membrane glycoprotein (M), ORF6 protei... Accession number: MZ287365 https://www.ncbi.nlm.nih.gov/nuccore/MZ287365 GenBank: Severe acute respiratory syndrome coronavirus 2 isolate SARS-CoV-2/human/UGA/MAKCHScov3/2020 ORF1ab polyprotein (ORF1ab), ORF1a polyprotein (ORF1ab), surface glycoprotein (S), ORF3a protein (ORF3a), envelope protein (E), membrane glycoprotein (M), ORF6 protein... Accession number: MZ287366 https://www.ncbi.nlm.nih.gov/nuccore/MZ287366 GenBank: Severe acute respiratory syndrome coronavirus 2 isolate SARS-CoV-2/human/UGA/MAKCHScov9/2021, complete genome Accession number: MZ287367 https://www.ncbi.nlm.nih.gov/nuccore/MZ287367 GenBank: Severe acute respiratory syndrome coronavirus 2 isolate SARS-CoV-2/human/UGA/MAKCHScov16/2020, complete genome. Accession number: MZ287368 https://www.ncbi.nlm.nih.gov/nuccore/MZ287368 GenBank: Severe acute respiratory syndrome coronavirus 2 isolate SARS-CoV-2/human/UGA/MAKCHScov25/2021, complete genome. Accession number: MZ287369 https://www.ncbi.nlm.nih.gov/nuccore/MZ287369 GenBank: Severe acute respiratory syndrome coronavirus 2 isolate SARS-CoV-2/human/UGA/MAKCHScov8/2021 ORF1ab polyprotein (ORF1ab), ORF1a polyprotein (ORF1ab), surface glycoprotein (S), ORF3a protein (ORF3a), envelope protein (E), membrane glycoprotein (M), ORF6 protein... Accession number: MZ287370 https://www.ncbi.nlm.nih.gov/nuccore/MZ287370 GenBank: Severe acute respiratory syndrome coronavirus 2 isolate SARS-CoV-2/human/UGA/MAKCHScov10/2021 ORF1ab polyprotein (ORF1ab), ORF1a polyprotein (ORF1ab), surface glycoprotein (S), ORF3a protein (ORF3a), envelope protein (E), membrane glycoprotein (M), ORF6 protei... Accession number: MZ287371 https://www.ncbi.nlm.nih.gov/nuccore/MZ287371 GenBank: Severe acute respiratory syndrome coronavirus 2 isolate SARS-CoV-2/human/UGA/MAKCHScov17/2020 ORF1ab polyprotein (ORF1ab), ORF1a polyprotein (ORF1ab), and surface glycoprotein (S) genes, partial cds; and ORF3a protein (ORF3a), envelope protein (E), membrane gl... Accession number: MZ287333 https://www.ncbi.nlm.nih.gov/nuccore/MZ287333 GenBank: Severe acute respiratory syndrome coronavirus 2 isolate SARS-CoV-2/human/UGA/MAKCHScov22/2021 ORF1ab polyprotein (ORF1ab), ORF1a polyprotein (ORF1ab), and surface glycoprotein (S) genes, partial cds; ORF3a protein (ORF3a) gene, complete cds; envelope protein (... Accession number: MZ287334 https://www.ncbi.nlm.nih.gov/nuccore/MZ287334 GenBank: Severe acute respiratory syndrome coronavirus 2 isolate SARS-CoV-2/human/UGA/MAKCHScov23/2021 ORF1ab polyprotein (ORF1ab) and ORF1a polyprotein (ORF1ab) genes, partial cds; surface glycoprotein (S), ORF3a protein (ORF3a), envelope protein (E), membrane glycopr... Accession number: MZ287335 https://www.ncbi.nlm.nih.gov/nuccore/MZ287335 Data has also been deposited in the Global Initial on Sharing All Influenza Data (GISAID’s EpiCoV database: http://www.gisaid.org) Zenodo: Whole-genome sequencing of SARS-CoV-2 in Uganda: implementation of the low-cost ARTIC protocol in resource-limited settings. https://doi.org/10.5281/zenodo.5055843. ^28^ This project contains the following extended data:
- Acknowledgments table for GISAID data Extended data are available under the terms of the Creative Commons Zero “No rights reserved” data waiver (CC0 1.0 Public domain dedication).
