# Supplementary material for: Whole-genome sequencing of SARS-CoV-2 in Uganda: implementation of the low-cost ARTIC protocol in resource-limited settings
Source: F1000Res. 2021 Jul 19;10:598. [Version 1] doi: 10.12688/f1000research.53567.1 (PMC8378408; doi:10.12688/f1000research.53567.1)
Supplement: Peer review history

# Peer review: Reviewer response for version 1

**DOI:** 10.5256/f1000research.56967.r89860

**Published:** 2021-08-19

**Authors:** Alan Christoffels

**Affiliations:** 1 South African Medical Research Council Bioinformatics Unit, South African National Bioinformatics Institute, University of the Western Cape, Cape Town, South Africa

Mboowa *et al. *describes the application of the ARTIC protocol for sequencing of SARS-COV-2 genomes and subsequently analysis in preparation for submission of data to GISAID.

**A few minor comments:**

**1. Abstract, Background**

 The context of this work points to the need for more adoption of WGS in Africa and therefore increase disease surveillance. One way of ordering the background paragraph could be: Start with the last sentence of that paragraph, remove the Wuhan sentence and then follow with the first sentence then mention that to date, x % of sars-cov-2 genomes submitted to GISAID were contributed from Africa. This data includes low quality viral genomes and a need for improved protocols for implementing WGS.

**2. Introduction, last paragraph**

 Replace UK variant and SA variant sentence with the acceptable nomenclature, "Alpha" and "Beta" variants respectively, so that this scientific record in F1000 assists in rectifying the public usage of terms.

**3. Methods section, study design**

 Is it standard protocol to archive SARS-CoV-2 biospecimens in the local biobank (IBRH3AU)? If not then it would be good to extend the sentence to add reproducibility for others wanting to do the same.

**4. Methods, seq and bioinformatics analysis section**

 Delete the first sentence that starts "These samples had been...". Edit the next sentence to read, "Metadata associated with the randomly selected 30 COVID-19 positive patient samples included the data of sample collection..."

**5. Discussion**

 This article is key to accelerating adoption of methods to respond to COVID-19. This focus of the paper was to show the success with using the ARTIC protocol. You could add a sentence in the results section after the phylogenetic analysis using iTOL to point readers to the option of using Nextstrain within the analysis process and the value of richer meta data in this context.

 Figures 1 and 2 have a title but no caption/description.

 Table 1: could you order the info in this table according to one of the columns? That would make reading the table a bit easier - maybe group by lineage? Or order by GC-content?

 Any comment on the 45.96 GC-content for MAKCHScov17/2020? Curious why the spike in comparison to the other samples.

Is the work clearly and accurately presented and does it cite the current literature?

Yes

If applicable, is the statistical analysis and its interpretation appropriate?

Not applicable

Are all the source data underlying the results available to ensure full reproducibility?

Yes

Is the study design appropriate and is the work technically sound?

Yes

Are the conclusions drawn adequately supported by the results?

Yes

Are sufficient details of methods and analysis provided to allow replication by others?

Partly

Reviewer Expertise:

Microbial bioinformatics, biobank information systems, data standards

I confirm that I have read this submission and believe that I have an appropriate level of expertise to confirm that it is of an acceptable scientific standard.

# Peer review: Reviewer response for version 1

**DOI:** 10.5256/f1000research.56967.r89859

**Published:** 2021-08-09

**Authors:** Anthony Mukwaya

**Affiliations:** 1 Department of Ophthalmology, Institute for Clinical, And Experimental Medicine, Faculty of Health Sciences, Linköping University, Linköping, Sweden

**Summary**

 The article by Mboowa *et al.*, evaluates the ARTIC protocol for Whole-genome sequencing of SARS-CoV-2 in Uganda. Authors test the protocol using previously collected qPCR positive COVID-19 samples, and they found that 93.3% of the samples generated readable genetic sequences. Of these samples, about 78% were of the A lineage, and 21% were of the B lineage. Authors show a relatedness of the isolates in the current study to other SARS-CoV-2 genomes available in GISAID. The study shows the feasibility of conducting the ARTIC protocol in a resource limited setting, thus allowing for more SARS-COV-2 WGS in the country. The idea behind the study is interesting, vital, and timely, given the role played by WGS in disease epidemiology. The article is properly written, the methods are robust, but few minor details need to be clarified. The comments below should be taken into consideration as they aim to further improve on the overall quality of the article.

**Minor corrections**

1. The following statement needs to be revised for accuracy: "Of the 131 Uganda full SARS-CoV-2 genomes analyzed in December 2020, 50 (38%)". I suspect authors meant "Of the 131 full SARS-CoV-2genomes analyzed in Uganda in December 2020…"?
2. I suggest a brief introduction of the SARS-CoV2 lineages/nomenclature be included in the introduction to guide the reader for example the "Pango lineages".
3. In the introduction, authors write "that this situation is very similar to almost all other African countries…" however, the "situation" being referred to is not properly problematized, thus needs to be explicitly defined in the preceding sentence (i.e. is it an issue with low testing rate or low/no sequencing rate or both)?
4. A brief introduction of ARTIC including the principle behind it would be good to summaries in the introduction, while highlighting the advantages of ARTIC over the currently available SARS-CoV-2 genome sequencing platforms.
5. Under the subheading study settings, the exact test used for COVID-19 diagnosis should be stated in this section as well. Under ethical consideration, please clearly state that "no patient information that can be used to identify an individual such as names and personal identification number etc. were used in the study".
6. Authors should elaborate on the details of the diagnostic protocol used for testing. For example, did all the amplification curves for all the three genes have to be below Ct 25 for a sample to be declared positive? What if only two genes had Ct values below 25, and the other gene above Ct 25, how was a such a sample classified? What would a Ct value of 26 mean? This being a diagnostic PCR, Interpretation of the diagnostic data should be clarified to indicate that many factors were taken into consideration, for example the total number of cycles run, and the Ct value range for interpretation, the positive control, and that of the internal control etc.
7. It is not clear if the samples used for sequencing were amplicons stored from the diagnostic process or if it was aliquots of RNA previously extracted during testing, or separate samples were collected from the individual specifically for this study. Please specify this in the methods.
8. Figures 1 and 2 need to be appropriately cited in the results main text.
9. In the recommendations, authors should recommend further testing of the ARTIC SARS-CoV-2 sequencing protocol using a much bigger sample size, different sample types, and preservation buffers, to draw more comprehensive conclusions.
10. Authors write that "the result of our study provides a thorough affirmation of carrying out whole genome sequencing for clinical SARS-CoV-2 samples in resource limited settings, thereby providing information to mitigate the impact of COVID-19 on our society". However, I think that it is important that the importance of sequencing on disease epidemiology be clearly stated as part of the study contribution.

Is the work clearly and accurately presented and does it cite the current literature?

Yes

If applicable, is the statistical analysis and its interpretation appropriate?

Yes

Are all the source data underlying the results available to ensure full reproducibility?

Yes

Is the study design appropriate and is the work technically sound?

Yes

Are the conclusions drawn adequately supported by the results?

Yes

Are sufficient details of methods and analysis provided to allow replication by others?

Partly

Reviewer Expertise:

Infection biology, functional genomics, Applied bioinformatics, molecular epidemiology, immunology, molecular biology, diagnostic methods, Angiogenesis, eye diseases

I confirm that I have read this submission and believe that I have an appropriate level of expertise to confirm that it is of an acceptable scientific standard.
